# Derivation of new human embryonic stem cell lines (Yazd1-3) and their vitrification using Cryotech and Cryowin tools: A lab resources report

**DOI:** 10.18502/ijrm.v17i12.5808

**Published:** 2019-12-30

**Authors:** Fatemeh Akyash, Somayyeh Sadat Tahajjodi, Ehsan Farashahi Yazd, Fatemeh Hajizadeh-Tafti, Fatemeh Sadeghian-Nodoushan, Jalal Golzadeh, Hassan Heidarian Meimandi, Harry Moore, Behrouz Aflatoonian

**Affiliations:** ^1^ Stem Cell Biology Research Center, Yazd Reproductive Sciences Institute, Shahid Sadoughi University of Medical Sciences, Yazd, Iran.; ^2^ Department of Reproductive Biology, School of Medicine, Shahid Sadoughi University of Medical Sciences, Yazd, Iran.; ^3^ Research and Clinical Center for Infertility, Yazd Reproductive Sciences Institute, Shahid Sadoughi University of Medical Sciences, Yazd, Iran.; ^4^ Abortion Research Center, Yazd Reproductive Sciences Institute, Shahid Sadoughi University of Medical Sciences, Yazd, Iran.; ^5^ Department of Biomedical Sciences, Centre for Stem Cell Biology, University of Sheffield, Western Bank, Alfred Denny Building, Sheffield S10 2TN, UK.; ^6^ Department of Advanced Medical Sciences and Technologies, School of Paramedicine, Shahid Sadoughi University of Medical Sciences, Yazd, Iran.

**Keywords:** Derivation, Human embryonic stem cells, Human foreskin fibroblast, Microdrop, Vitrification.

## Abstract

**Background:**

Cell banking of initial outgrowths from newly derived human embryonic stem cells (hESCs) requires an efficient freezing method. Vitrification is used for the preservation of gametes and early embryos in assisted reproduction techniques (ART). Moreover, vitrification was applied for cryopreservation of hESCs using open pulled straws.

**Objective:**

To derive and characterize new hESC lines and then use Cryotech and Cryowin tools for their vitrification.

**Materials and Methods:**

Human ESC lines were generated in a microdrop culture system using mouse embryonic fibroblasts (MEFs) as the feeder layer; this was later scaled up using both MEFs and Yazd human foreskin fibroblasts batch 8 (YhFF#8). To bank the cell lines, master cell banks of 100 Cryotech and Cryowin tools were produced for each individual cell line using the vitrification method; flasks of hESC lines were also cryopreserved using a conventional slow-freezing method.

**Results:**

The pluripotency of cell lines was assessed by their expression of pluripotency-associated genes (*OCT4/POU5F1*,* NANOG*, and* SOX2*) and markers such as SSEA4, TRA-1-60, and TRA-2-49. Their *in vitro* capacity to differentiate into germ layers and germ cells using embryoid body (EB) formation and monolayer culture was assessed by screening the expression of differentiation-associated genes. The chromosomal constitution of each hESC line was assessed by G-banding karyotyping.

**Conclusion:**

Cryotech and Cryowin tools used to vitrify new hESCs at an early stage of derivation is an efficient method of preserving hESCs.

## 1. Introduction

Stem cells have been known for a long time, but the generation of embryonic stem cells (ESCs) altered the concept of what constitutes a stem cell (1). Using immunosurgery (2), mouse ESCs (mESCs) were first derived in 1981 (3, 4). Later, other groups made unsuccessful attempts to generate human ESCs (hESCs) (5), followed by generation of ESCs in primates (6, 7), and eventually, in 1998, hESCs were derived (8). This coincided with the advent of their putative counterpart, human embryonic germ cells (hEGCs) (9). Typical hESC morphology is an initial characteristic that helps in the derivation process. The morphology of hESCs is very similar to pluripotent embryonal carcinoma cells (ECCs), with a high ratio of nuclei to cytoplasm, pale nuclei, and prominent nucleoli (10). Four major factors play a critical role in the hESC derivation process. (a) The source and stage of pre-implantation embryos from cleavage (11) to blastocyst (8). Recently, it was shown that embryos obtained following *in vitro* twinning can be used for the generation of hESC-like cells; however, attempts to establish a cell line have yet to succeed (12). (b) The method of derivation used, such as inner cell mass (ICM) isolation using immunosurgery (13), laser-assisted ICM biopsy (14), blastomere biopsy (15), mechanical isolation of the ICM (16), and whole zona-free blastocyst culture (13, 17). (c) Different sources of feeder layer, from mouse embryonic fibroblasts (MEFs) (13) to human derived feeders, such as human foreskin fibroblasts (HFFs) (12, 18), human fetal gonadal fibroblasts (HFGFs) (13), human endometrial-derived fibroblasts (19), and human cumulus cells (hCCs) (20). (d) The scale of cell culture used, i.e., either an open (13, 17) or a microdrop system (13).

Following their initial derivation, hESCs must be cryopreserved and expanded for further characterization of specific gene and marker expression to assess their undifferentiated status (13). In addition, their capacity to differentiate into the three germ layers (ectoderm, mesoderm, and endoderm) and germ cells, to demonstrate their pluripotency, should be evaluated, either by *in vitro* embryoid body (EB) formation or by in vivo teratocarcinoma formation, to investigate further differentiation potential (21). The chromosome content of the cell line is another issue that can be evaluated by G-binding or the CGH-array method (22). One of the challenges in banking any cell type is the method of freezing used. The use of an ideal method for cryopreservation can improve the survival rate and proliferative capacity of post-thawed hESCs (23). Studies have demonstrated that fewer than 5% of hESCs survived an equilibrium slow-freezing procedure using 10% dimethylsulphoxide (DMSO) in fetal calf serum; in contrast, high viability among hESCs was reported when using a vitrification procedure for the cell lines using an “open pulled-straw” method with a small volume of cells (13). Vitrification is a state-of-the-art method used for the freezing of a small number of cells, including gametes and embryos, and is used for the cryopreservation of hESCs using an open pulled-straw method (13). Vitrification is also a good choice of method to use shortly after the derivation of hESCs that are in urgent need of cell line preservation (23).

Here, we report the vitrification of new outgrowths to save newly derived hESC lines (Yazd1-3) using Cryotech and Cryowin tools. Whole, zona-free blastocysts were cultured on an MEF feeder layer in microdrop culture. The purpose of this study was first to derive and characterize new hESC lines and then using Cryotech and Cryowin tools for their vitrification (although this method was not compared with a conventional open pulled-straws method).

## 2. Materials and Methods

Chemicals were purchased from Sigma Aldrich (Poole, UK). Culture media and supplements were purchased from Invitrogen and Gibco (UK), unless otherwise stated.

### Embryo culture

The vitrified donated embryos (n = 10) were warmed as described elsewhere (24) and cultured in a microdrop system with G series medium (version III; Vitrolife) plus 5% human serum albumin (Vitrolife) till getting to the blastocyst stage. The fresh donated embryos were cultured in the same culture medium for in vitro blastocyst development.

### Preparation of the microdrops of feeders

MEFs were derived from Naval Medical Research Institute (NMRI) mouse embryos according to ethical guidelines relating to animals and cultured as described elsewhere (25). Briefly, 13 days after the appearance of the vaginal plug, fetuses were recovered from the uterus and their heads, spinal cords, and livers were removed. Following enzymatic and mechanical treatment, the resulting cell suspension was transferred to a T25 tissue culture flask containing Dulbecco's Modified Eagle's Medium (DMEM), 10% fetal bovine serum (FBS), and antibiotics, then incubated at 37°C in 5% ${\mathrm{CO}}_{2}$ in air. Yazd HFFs batch 8 (YhFF#8) were isolated and expanded from neonatal human foreskin tissues after obtaining fully informed written consent, according to the guidelines of the Shahid Sadoughi University of Medical Sciences Ethical Committee (ethics committee reference number: IR.SSU.REC.1394.103; Aflatoonian *et al.*, unpublished data). MEFs and YhFFs#8 were mitotically inactivated using Mitomycin-C (by adding 2 mg Mitomycin-C to 200 ml DMEM/10% FBS) for 2 and 4 hrs, respectively. After washing three times with Dulbecco's phosphate buffered saline (D-PBS), cells were detached using 1 x trypsin/EDTA, centrifuged at 1000 rpm for 5 minutes, and then re-plated in 50 µl drops onto dishes (Falcon, USA) at a concentration of 3,500 cells per 50 µl and covered with mineral oil (Irvine Scientific, USA). The following day the microdrop medium of feeders was replaced with KOSR/HES medium (containing KnockOut DMEM, KnockOut Serum Replacement, L-glutamine/β mercaptoethanol, bFGF, non-essential amino acids, and antibiotics) and left for 2 hrs for conditioning prior to use. Each dish was plated with seven microdrops of feeder layer.

### Generation of Yazd hESC lines (Yazd1-3) using the microdrop method

To obtain intact zona-free blastocysts, and based on the grade of each individual blastocyst, embryos were either cultured to fully hatched blastocyst stage or were treated with pronase (2 mg/ml; Calbiochem, USA) to lyse the zona pellucida. The intact zona-free blastocysts were plated onto the microdrops of MEF feeders. Yazd1 cells were initially generated using VitroHES culture medium (ready-to-use from Vitrolife), Yazd2 cells were derived and expanded in KOSR/HES medium, while for Yazd3 cells were, the blastocysts were initially cultured in DMEM/10% FBS overnight, and the following day the medium was exchanged for KOSR/HES medium. Small modifications to the culture conditions for the derivation of each individual Yazd hESC line (Yazd1-3) are shown in Table I.

All three cell lines were derived using intact embryo co-culture on a microdrop of MEF feeders. All lines were later expanded on both MEFs (initially) and Yazd HFFs (YhFF#8). Yazd1 cells were generated from a fresh embryo, which was cultured to the blastocyst stage (grade 3CB) in VitroHES medium. Yazd2 cells were derived from a frozen-warmed embryo, which was allowed to develop to the early cavity stage (grade 1) on KOSR/HES medium. Yazd3 cells were generated from an embryo which lost its zona during the freezing and thawing process and was allowed to develop to the blastocyst stage (grade 2). All cell lines were expanded using KOSR/HES medium. Yazd1 and Yazd2 hESC lines contained normal karyotypes (46, XX and 46, XY, respectively); however, the Yazd3 karyotype was initially a triploid 69, XXY.

### Expansion and EB formation of Yazd hESC lines (Yazd1-3)

**Figure 1:**
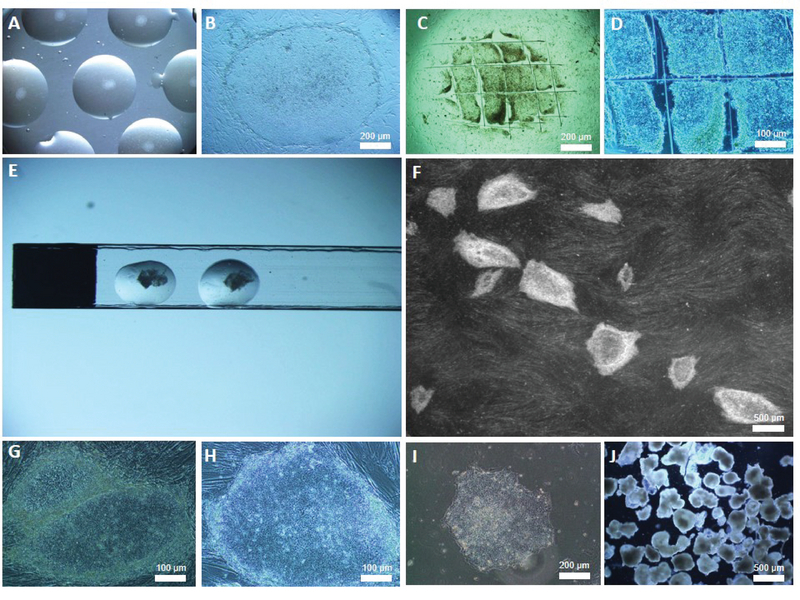
Culture of Yazd1-3 hESC lines at different scales on MEFs and YhFFs#8. The one drop-one colony method was used for expansion of the Yazd hESC lines (A, B). For the passage procedure, following enzymatic treatment, the medium was replaced with KOSR/HES medium and colonies were cut into smaller pieces (C, D). Cryotech and Cryowin tools were used for the vitrification of Yazd hESCs from the initial derivation steps. In this figure, each drop contains two relatively large pieces of hESC colonies (E). Yazd1-3 hESC lines were expanded using larger scale culture methods, such as central well dishes and tissue culture flasks (F). Both MEFs (A, B, and H) and YhFFs#8 (F, G) were used for the expansion of Yazd1-3 hESC lines. Matrigel was applied with KOSR/HES-conditioned medium for the feeder-free culture of colonies (I). The EB formation procedure was similar to passaging, in which small pieces of colonies were plated in non-adherent culture dishes with EB medium (J).

To establish the Yazd1-3 hESC lines following initial hESC outgrowth formation, the cells were mechanically passaged using a mouth pipette and pulled Pasteur pipettes. To detach the colonies, they were first treated with collagenase type IV (1 mg/ml) at 37°C in 5% ${\mathrm{CO}}_{2}$ in air for 8-10 min. A one drop-one colony method was used for the expansion of the Yazd hESC lines (Figure 1A and B). Following enzymatic treatment, the medium was replaced with KOSR/HES medium, colonies were cut into smaller pieces (Figure 1C and D) gently by pulled glass Pasteur pipette were plated either onto the new feeders (in different scales) with KOSR/HES medium (Figure 1A and F). Both YhFF#8 (Figure 1G) and MEFs (Figure 1H) were used for the expansion of Yazd1-3 cell lines. To prevent hESCs differentiating, daily feeding with fresh medium was performed by replacing half the medium. Occasionally, Yazd3 colonies appeared morphologically different from the normal flattened shape, becoming rounder and forming clusters, possibly due to the development of progenitor cells. To prevent further differentiation, these colonies were treated with dispase (10 mg/ml in KOSR/HES). Briefly, colonies were washed twice with pre-warmed PBS and then cut into small pieces. The PBS was replaced with dispase solution following incubation for 2 minutes at 37°C. The small pieces of colonies were washed in three pre-warmed PBS drops, and then plated onto new microdrops containing feeders, each drop one colony. For the feeder-free culture of colonies, Matrigel was applied together with KOSR/HES-conditioned medium (Figure 1I). The EB formation procedure (Figure 1J) was similar to passaging, in which small pieces of colonies were plated in non-adherent culture dishes with EB medium (KOSR/HES without bFGF).

### Cryopreservation and thawing of Yazd hESC lines (Yazd1-3)

Two different methods were applied for freezing the Yazd1**-**3 hESC lines, according to the scale of the culture. Colonies within the microdrops for master cell-banking were cryopreserved using Cryotech and Cryowin tools (Cryotec, Cryotech, Japan and Cryowin, TechWin, Iran; Figure 1E) for the vitrification method (13). For larger scale cultures, such as central well dishes or tissue culture flasks (Figure 1F), at later passages colonies were frozen using a slow-freezing solution consisting of 10% DMSO in FBS. In summary, for vitrification, small pieces (larger than pieces usually used for passaging) of hESC colonies were scraped off and placed into a small drop of KOSR/HES medium. Then, colonies were transferred into homemade vitrification solutions 1 and 2 (VS1 and VS2) containing ethylene glycol and DMSO, first at 10%, then at 20%, respectively, in basal medium (DMEM, 20% FBS, and non-essential amino acids). Small colonies were drawn onto the Cryotech and Cryowin tools, plunged into liquid nitrogen, and then transferred to long cryovials, which were sealed with a cap located in the cryocane and placed in a liquid nitrogen storage container. For thawing, a Cryotech or Cryowin was taken from the liquid nitrogen container tank and then placed into a drop of KOSR/HES medium. The cells were washed five times by transferring them through more drops before being placed onto microdrops containing feeders in KOSR/HES medium and incubated at 37°C at 5% ${\mathrm{CO}}_{2}$ in air.

### Immunofluorescent localization (IF) of pluripotency markers

The identification of pluripotency-associated cell markers was carried out using IF localization, as previously described (26). In summary, Yazd1-3 hESC colonies were washed with PBS containing 1% FBS before being fixed in cooled methanol ( - 20°C) for 15 minutes. Samples were washed twice for 5 min in D-PBS containing 1% FBS and then incubated overnight at 4°C with primary antibodies (SSEA4, TRA-1-60, and TRA-2-49; kindly gifted by Professor Peter W. Andrews from the Centre for Stem Cell Biology, Sheffield University, UK) diluted (1:10) in D-PBS. Samples were then washed twice in PBS and incubated with appropriate secondary antibodies for 1 hr at 37°C (see Table II). Preparations were covered with mounting medium (Vectashield, Vector Laboratories, USA) and examined microscopically using fluorescence with appropriate excitation optics on an inverted microscope (Olympus IX-71). Experiments were performed in triplicate.

### RNA isolation, cDNA production, and reverse transcription PCR

Undifferentiated colonies and spontaneously differentiated EBs following different culture periods (4, 7, and 14 days) and differentiated colonies in the monolayer culture system (after 7 days) were collected. The resulting pellets were suspended in 1 mL TRI reagent (Sigma) and total RNA was extracted according to the standard protocol supplied by the manufacturer. Total RNA was treated with DNase I (RNase-free Kit; Fermentas, Germany) to remove genomic DNA. First-strand cDNA synthesis was performed using the Superscript II reverse transcriptase system (Fermentas, Germany). PCR was performed using the prepared cDNA and primers for different genes (Table III) by Platinum Blue PCR Super Mix (Invitrogen, UK) in triplicate. PCR cycling conditions were as follows: 5min at 95°C for initial denaturation, followed by 40 cycles of 45 sec at 95°C, 45 sec at 59°C-65°C (see Table III), and 45 sec at 72°C.

### Karyotype analysis of Yazd hESC lines (Yazd1-3)

To investigate the chromosomal content of Yazd1-3 hESC lines, the karyotype of ten cells in metaphase of each Yazd cell line was determined by a standard G-banding procedure. Briefly, cells were cultured in flasks without feeder for 5 to 6 days to form mesenchymal-like cells (27). Following treatment with colchicine (10 µg/ml), harvested Yazd hESC-derived cells were stained using a standard G-banding technique. G-bandings were analyzed under light microscopy (Axiophot, Ziess, Germany) using applied spectral imaging software.

### *In vitro* differentiation of Yazd hESC lines (Yazd1-3)

*In vitro* differentiation of Yazd1-3 hESC lines was performed as previously described (13), using EB formation and gene expression profile assessment after 4, 7, and 14 days for mesoderm, endoderm, ectoderm, and germ-cell development. The same was done for spontaneously differentiation in the monolayer culture with DMEM/10% FBS (after 7 days).

**Table 1:** A summary of the derivation information for the Yazd1-3 hESC lines


**Yazd hESC line**	**Frozen or fresh embryo**	**Embryo grade and stage**	**Pronase treatment**	**Feeder for initial derivation**	**Feeder for expansion**	**Medium for initial derivation**	**Initial karyotype**
Yazd1	Fresh	3CB full blastocyst	+ MEF	MEF/ YhFF#8	VitroHES (Vitrolife)	46, XX
Yazd2	Frozen	1 early blastocyst	+ MEF	MEF/ YhFF#8	KOSR/HES	46, XY
Yazd3	Frozen	2 blastocyst	- MEF	MEF/ YhFF#8	DMEM/10% FBS	69, XXY

**Table 2:** List of primary and secondary antibodies used for immunofluorescent (IF) localization


**Primary antibody**			**Secondary antibody**		
**Name**	**Dilution**	**Catalog number**	**Type**	**Dilution**	**Catalog number**
TRA-1-60		Rabbit anti-mouse IgG+IgM+IgA H&L (FITC)	abcam ab8517
MC813-70 (SSEA-4)			
TRA-2-49	<brow>-4</erow> 1:10	<brow>-4</erow> Kindly gifted by Prof. Peter W. Andrews (CSCB, Sheffield, UK)	<brow>-2</erow> Rabbit anti-mouse IgG H&L (TR)	<brow>-4</erow> 1:200	<brow>-2</erow> abcam ab6726

**Table 3:** List of primers used for RT-PCR, showing speciﬁcations


	**Gene**	**Forward primer (5'-3')**	**Reverse primer (5'-3')**	**Product size (bp)**
	*NANOG *(NM-024865.3)	CCCCAGCCTTTACTCTTCCTA	CCAGGTTGAATTGTTCCAGGTC	97
	*POU5F1* (NM-002701.5)	GGTTGAGTAGTCCCTTCGCA	TAGCCAGGTCCGAGGATCA	174
<brow>-3</erow> Pluripotency	*SOX2* (NM-003106.3)	AGGACTGAGAGAAAGAAGAGG	GAGAGAGGCAAACTGGAATC	163
	*PAX6* (NM-000280.4)	AGATTCCTATGCTGATTGGTGAT	AGGAGGAAGTGTTTTGCTGGA	139
<brow>-2</erow> Ectoderm	*TUBB3* (NM-006086.3)	ACCAGATCGGGGCCAAGT	GGCACGTACTTGTGAGAAGAGG	142
	*DES* (NM-001927.3)	GTGCATGAAGAGGAGATCCGT	ATGTTCTTAGCCGCGATGGT	143
<brow>-2</erow> Mesoderm	*TBXT* (NM-003181.3)	GGCGCGAGAACAGCACTA	CCAAGACTGTCCCCGCTC	117
Endoderm	*SLC2A1* (NM_006516.2)	TTGGCTCCGGTATCGTCA	CTCAGATAGGACATCCAGGGTA	172
	*VASA* (NM-024415.2)	ACAGATGCTCAACAGGATGTTCC	CCCTTTCTGGTATCAACTGATGCA	119
<brow>-2</erow> Germ-cell	*DAZL* (NM-001351.3)	CCTTGTCACCCGCTCTTG	ATTTGCAGTAGACATGATGGCG	183
Housekeeping	*B2M* (NM-004048.2)	AGATGAGTATGCCTGCCGTG	TGCGGCATCTTCAAACCTC	106

### Ethical considerations

Sixteen fresh (n = 6) and frozen (n = 10) supernumerary embryos were donated for research by infertile couples after they had provided full, written, informed consent according to the Shahid Sadoughi University of Medical Sciences Ethical Committee (ethics committee reference number: IR.SSU.REC.1394.104). For research purposes, all embryos were coded to ensure patient anonymity.

## 3. Results

### Generation of Yazd1-3 hESC lines

**Figure 2:**
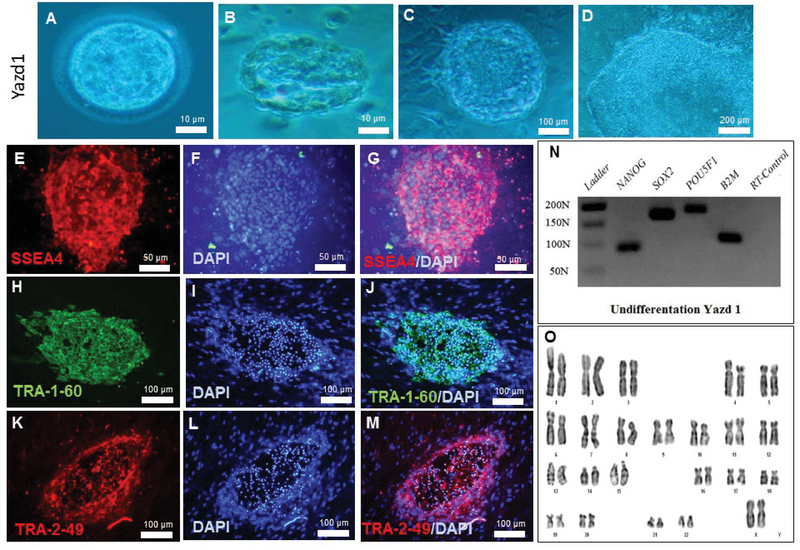
Derivation and characterization of the Yazd1 hESC line. The Yazd1 cells were produced from a donated, fresh, grade 3CB full blastocyst (A). The blastocyst stage embryo was plated onto a microdrop of MEFs (B). The first outgrowth of cells was observed after two days (C); following eight passages, colonies with typical hESC morphology (D) were visible. The expression of pluripotency markers SSEA4 (E-G), TRA-1-60 (H-J), and TRA-2-49 (K-M) was assessed by IF staining. The expression of pluripotency genes *NANOG*, *SOX2*, and *OCT4/POU5F1 *(N) was evaluated using RT-PCR of undifferentiated Yazd1 hESCs. A normal, 46, XX karyotype for the Yazd1 hESC line (O) was detected by G-binding. The latest passage of Yazd1 for characterization was passage 7+5+10.

**Figure 3:**
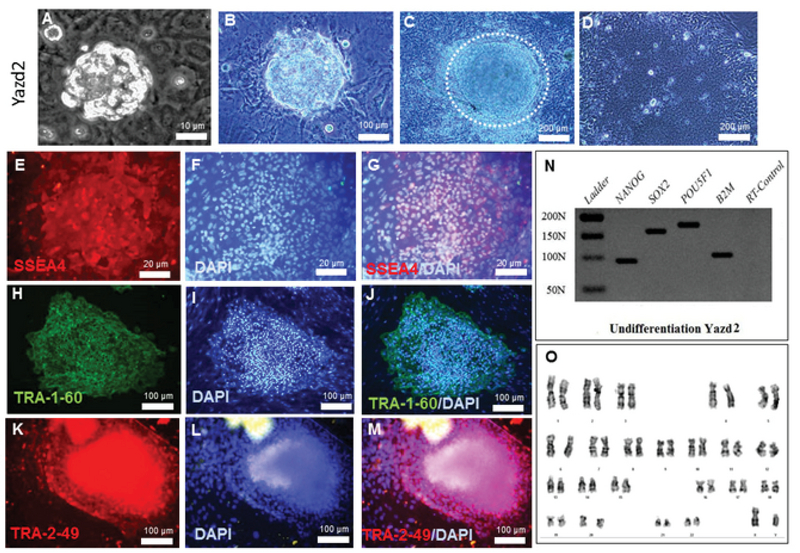
Derivation and characterization of the Yazd2 hESC line. The Yazd2 cells were generated from an early blastocyst stage (grade 1) embryo plated onto a microdrop of MEFs (A). The first outgrowth after two (B) and three days (C). After several passages, hESC colonies were distinguished (D). The positive expression of the pluripotency markers SSEA4 (E-G), TRA-1-60 (H-J), and TRA-2-49 (K-M) was tested using IF. RT-PCR data showed expression of the pluripotency genes, *NANOG*, *SOX2*, and *OCT4/POU5F1 *(N) in undifferentiated cells. A normal 46, XY karyotype for the Yazd2 hESC line (O) was detected using G-binding. Yazd2 is still in culture with the latest passage number being P8+38+58. The characterization of Yazd2 was performed after P8+38+10.

**Figure 4:**
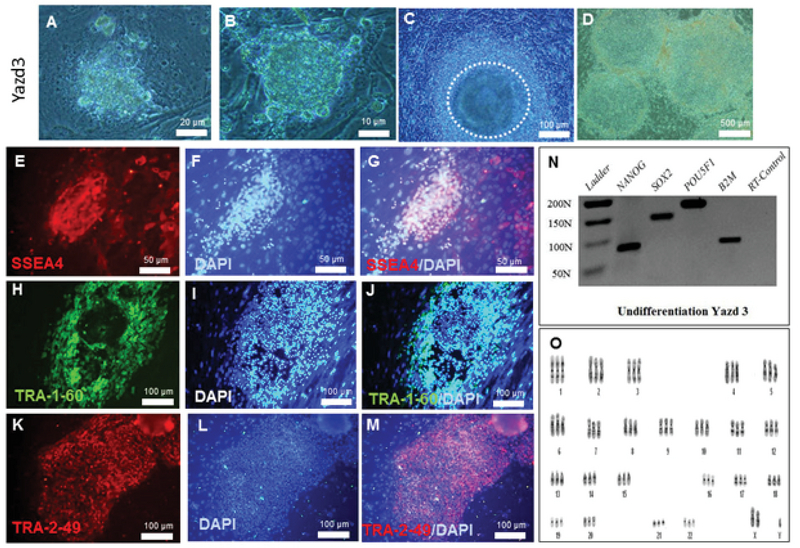
Derivation and characterization of the Yazd3 hESC line. The Yazd3 cells were derived from a zona-free, grade 2, blastocyst stage embryo, which was plated onto a microdrop of MEFs (A). The first outgrowth after two (B) and five days (C). hESC colonies of Yazd3 in later passages (D). The positive expression of the pluripotency markers SSEA4 (E-G), TRA-1-60 (H-J), and TRA-2-49 (K-M) was tested using IF. RT-PCR data showed the expression of the pluripotency genes *NANOG*, *SOX2*, and *OCT4/POU5F1 *(N). An abnormal, triploid 69, XXY karyotype for the Yazd3 hESC line (O) was shown using G-binding. The Yazd3 cells were frozen down after passage number 169. The karyotype of Yazd3 was assessed during passages 4, 32, and 89. Characterization of Yazd3 was done after passage 68.

In the current study, sixteen fresh (n = 6) and frozen (n = 10) donated surplus human embryos were used to generate Yazd1-3 hESC lines using a microdrop culture system (13) (Figure 1A and B) and MEFs as feeder layer cells. The pre-implantation human embryos (Figure 2A and 3A) were used as described above following pronase treatment (for Yazd1 and Yazd2) in a 50 μl microdrop co-culture system (Figure 1A and B). The Yazd1 cell line was produced from a fresh 3CB full blastocyst donated for stem cell research (Figure 2A), which was plated onto a microdrop of MEFs (Figure 2B). After two days, the outgrowth of cells was observed (Figure 2C). Following eight passages, colonies with a typical hESC morphology were obtained (Figure 2D). The Yazd1 cell line was derived and initially expanded using VitroHES (Vitrolife) culture medium. For the generation of the Yazd2 cell line, a frozen-warmed embryo, which had been donated for stem cell research was developed to the early blastocyst stage (grade 1) *in vitro*, the zona pellucida was lysed as described above, and plated onto a microdrop of MEFs (Figure 3A) in KOSR/HES medium. The first outgrowths after two and three days were as shown in Figure 3B and C, respectively. As indicated in Figure 3C, a population of cells were overgrown, resembling putative hEGC clusters (28), which were distinguished from these areas after several passages of hESC colonies (Figure 3D). To derive Yazd3 cells, a frozen embryo donated for stem cell research was thawed. During thawing the zona pellucida was destroyed and the zona-free embryo was allowed to develop to blastocyst stage (grade 2) *in vitro*, plated onto a microdrop of MEFs (Figure 4A), and then incubated overnight in DMEM/10% FBS. On the following day attachment of the embryo onto the feeder layer and the medium was replaced with KOSR/HES. The first outgrowths after two and five days were as shown in Figure 4B and C, respectively. Following passaging, as previously described (13) and explained above, for Yazd2 cells a compact area (Figure 4C) was distinguished from other parts of the colony from which, in later passages, typical hESC colonies were generated (Figure 4D). Summary information of modifications to the techniques used for derivation of Yazd1-3 hESC lines is shown in Table I.

### Establishment of Yazd1-3 hESC lines

All Yazd cell lines (1-3) were initially derived and expanded on MEFs (Figure 1 A, B, and H). Later, YhFFs#8 (Figure 1F and G) and Matrigel (Figure 1I) were applied to maintain undifferentiated Yazd hESCs in culture. Half of the KOSR/HES medium was replaced with fresh medium each day. Non-adherent petri-dishes with EB medium were used to produce EBs (Figure 1J) to induce spontaneous differentiation *in vitro *(13).

Yazd1 hESCs (passage number 7+5+10) showed positive expression of pluripotency associated markers, SSEA4 (Figure 2E-G), TRA-1-60 (Figure 2H-J), and TRA-2-49 (Figure 2K-M), as assessed using an IF technique. Undifferentiated Yazd1 cells expressed the pluripotency genes *NANOG*, *SOX2*, and *OCT4/POU5F1 *(Figure 2N) determined by RT-PCR and contained normal 46, XX karyotypes (Figure 2O). Yazd2 hESCs (passage number 8+38+10) displayed expression of pluripotency-associated markers such as SSEA4 (Figure 3E-G), TRA-1-60 (Figure 3H-J), and TRA-2-49 (Figure 3K-M) and genes such as *NANOG*, *SOX2*, and *OCT4/POU5F1 *(Figure 3N), as assessed by IF and RT-PCR, respectively. The initial karyotype of Yazd2 hESCs was normal, 46, XY, as revealed by a G-binding procedure (Figure 3O). The undifferentiated Yazd3 hESC colonies (passage number 68) were characterized by SSEA4 (Figure 4E-G), TRA-1-60 (Figure 4H-J), and TRA-2-49 (Figure 4K-M) using IF, and *NANOG*, *SOX2*, and *OCT4/POU5F1* using RT-PCR (Figure 4N). Yazd3 was an abnormal hESC line with a karyotype of 69, XXY in its early passages (passage number 8; Figure 4O).

### *In vitro* differentiation of Yazd1-3 hESC lines

**Figure 5:**
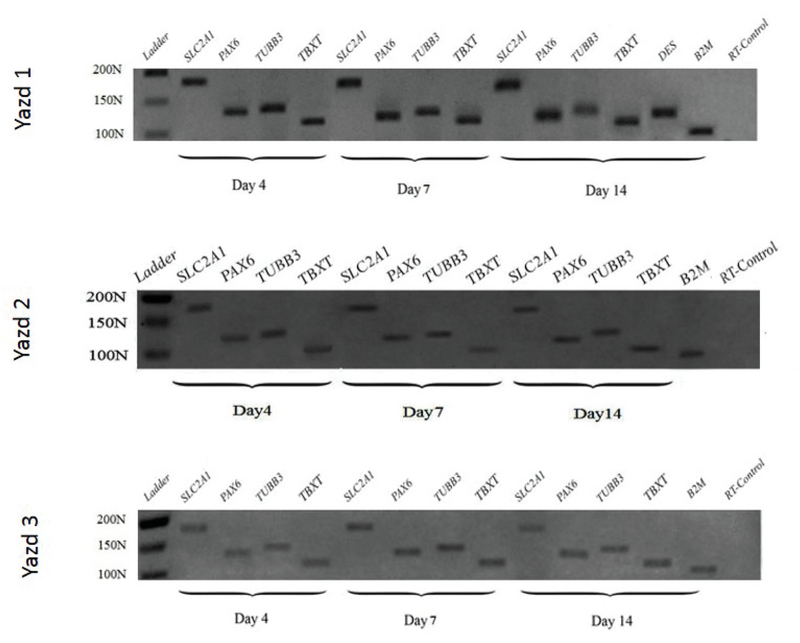
RT-PCR for the three embryonic germ-layer markers. Differentiation of Yazd1-3 hESC lines into the three embryonic germ layers (ectoderm: *PAX6*,* TUBB3*; mesoderm: *DES*,* TBXT*; endoderm: *SLC2A1*) using EB formation after 4, 7, and 14 days.

**Figure 6:**
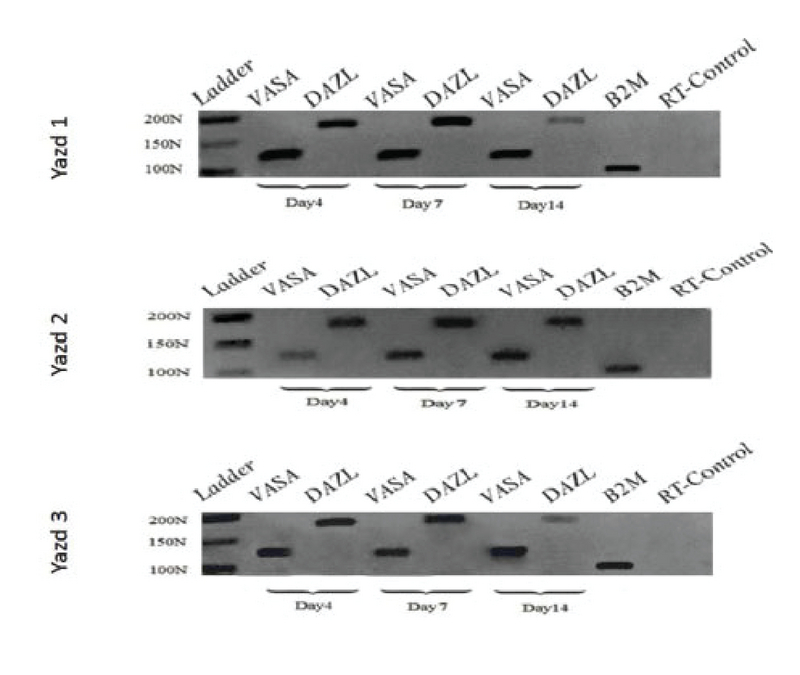
RT-PCR for germ-cell markers. Differentiation of Yazd1-3 hESC lines to germ cells (*VASA *and* DAZL*) using EB formation after 4, 7, and 14 days.

**Figure 7:**
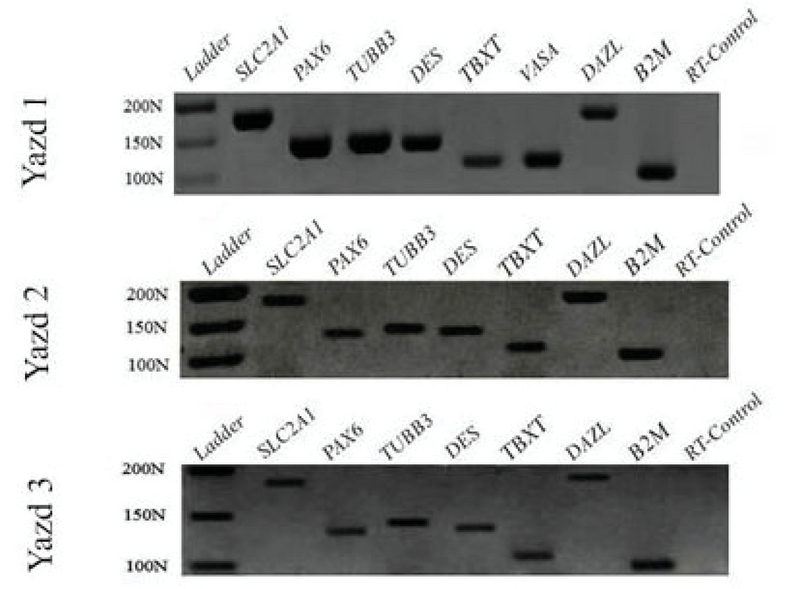
RT-PCR for three embryonic germ-layer/germ-cell markers. Differentiation of Yazd1-3 hESC lines to the three embryonic germ layers (ectoderm: *PAX6*,* TUBB3*; mesoderm: *DES*,* TBXT*; endoderm: *SLC2A1*) and germ cells (*VASA *and* DAZL*) using monolayer culture after 7 days.

The pluripotent differentiation capacity of the first three Yazd hESC lines (Yazd1-3) was proven *in vitro* using different EB formations (Figure 5 and Figure 6) and monolayer cultures (Figure 7), with assessment of the expression of specific genes for the three germ layers and germ cells. Differentiated cells expressed specific genes of the three embryonic germ layers (*SLC2A1*,* PAX6*,* TUBB3*,* DES*, and *TBXT*) and germ cells (*VASA* and *DAZL*).

## 4. Discussion

This study reports the derivation of Yazd1-3 hESC lines, initially using an MEF feeder layer in a microdrop culture system, and the banking of these cell lines using Cryotech and Cryowin tools for vitrification, which has started since 2008. Later Yazd hESCs were expanded on YhFF#8 while establishment and characterization.

Following the generation of Shef lines using the microdrop culture system, we have applied this method for the derivation of new Yazd hESC lines (13). In recent studies, the efficiency of hESC derivation was reported to be more than 50% (29). In this study, however, three new hESC lines (Yazd1-3) were generated from sixteen fresh and frozen embryos, representing a success rate of 18.75%. This difference could be due either to the source of the embryos or the culture conditions, which in our case involved conventional KOSR/HES medium with MEFs as a feeder layer. The success rate for hESC generation from fresh embryos in this study was about 16.66%, whereas it was 20% for hESC lines derived from frozen embryos. As far as we know, this is the first report to describe the vitrification of hESCs using Cryotech and Cryowin tools; however, no comparisons were made between the efficiency of this method with the conventional open pulled straws method. This new method is applicable for the preservation of early outgrowths in early passages of pluripotent stem cells, and in our experience: hESCs. The new Yazd1-3 hESC lines were characterized by specific pluripotency markers, such as SSEA-4, TRA-1-60, and TRA-2-49, and pluripotency genes, including *NANOG*, *SOX2*, and *OCT4/POU5F1*. Furthermore, the differentiation potential of the cell lines in EB formation and monolayer culture was determined using RT-PCR for the three embryonic germ layers and germ-cell-specific genes.

The use of a microdrop culture system was first reported in 1994 for the culturing of bovine ESC (30). In an open culture system, secreted growth factors may be lost or diluted, whereas it has been demonstrated that a combination of a co-culture and a microdrop system had the highest rates of blastocyst formation and hatching in comparison with an open co-culture system (31). Wakayama and colleagues (32) derived mESC lines using an efficient micro-scale method. Single blastomeres were co-cultured with MEFs in individual wells of 96-well plates. Following that, Aflatoonian and co-workers generated hESC lines using a microdrop culture system (13).

In assisted reproductive techniques (ART), a laser is commonly used to assist with different processes, such as assisted hatching (33) and blastomere and trophectoderm (TE) biopsies (34). In addition, laser-assisted technology has been used for ICM biopsies in mice (35, 36) and humans (14, 22, 37, 38, 39). In the present study, no ICM isolation methods, such as immunosurgery and laser-assisted biopsy, were used. The Yazd1-2 hESC lines were derived from blastocyst and early cavity embryos respectively, which were extruded from the zona pellucida by pronase treatment. It should be noted; however, that pronase is derived from bacteria and is therefore not suitable for the derivation of clinical grade hESCs in the future (14). The Yazd3 hESC line was derived from a zona-free embryo following the thawing process. While the ICM is known to be the usual source for hESC derivation, the development of hESCs from single blastomeres of cleavage- and morula-stage embryos (12, 40, 41, 42) has also been reported, but these methods have not been used routinely.

To avoid animal contamination following the co-culture of hESCs with MEFs, xeno-free derivation of hESC lines can be achieved by using human feeders including HFFs (18), HFGFs (13), human endometrial-derived fibroblasts (19), hESC-derived feeder (EDF), and human placental fibroblast feeders (43). In this regard, hESCs derived fibroblast-like cells conditioned medium demonstrated to support undifferentiated hESC proliferation under feeder-free conditions (44).

Herein, MEFs were used for the initial derivation of Yazd1-3 hESC lines, followed by expansion of the cell lines on MEFs and YhFFs#8. Mesenchymal stem/stromal cells have been derived from Yazd hESC lines (27), although hESC-derived MSCs have yet to be used as feeders.

The Yazd cell lines, which were characterized as described above and have been used during workshops (45), are available for other research groups and companies to use, according to Yazd Reproductive Sciences Institute rules and laws, as the owner of the cell lines.

## 5. Conclusion

New hESC lines (Yazd1-3) were derived on microdrops of an MEF feeder layer, without immunosurgery, using whole blastocyst culture. Following expansion of the cell lines, YhFFs#8 were used as a xeno-free condition with KOSR/HES. This study is the first report of Cryotech and Cryowin tools being used for the vitrification of hESCs in the very early stages of derivation. Further studies are in progress to evaluate the efficiency of this method compared with other techniques and tools.

##  Conflict of Interest

The authors declare no conflicts of interest.

## References

[1] Evans M. (2009). Isolation and Maintenance of Murine Embryonic Stem Cells. *Essentials of Stem Cell Biology*.

[2] Solter D., Knowles B. B. (1975). Immunosurgery of mouse blastocyst.. *Proceedings of the National Acadamy of Sciences of the United States of America*.

[3] Evans M. J., Kaufman M. H. (1981). Establishment in culture of pluripotential cells from mouse embryos. *Nature*.

[4] Martin G. R. (1981). Isolation of a pluripotent cell line from early mouse embryos cultured in medium conditioned by teratocarcinoma stem cells. *Proceedings of the National Acadamy of Sciences of the United States of America*.

[5] Bongso A., Fong C., Ng S., Ratnam S. (1994). Fertilization and early embryology: Isolation and culture of inner cell mass cells from human blastocysts. *Human Reproduction*.

[6] Thomson J. A., Kalishman J., Golos T. G., Durning M., Harris C. P., Becker R. A., Hearn J. P. (1995). Isolation of a primate embryonic stem cell line. *Proceedings of the National Acadamy of Sciences of the United States of America*.

[7] Thomson J. A., Kalishman J., Golos T. G., Durning M., Harris C. P., Hearn J. P. (1996). Pluripotent cell lines derived from common marmoset (Callithrix jacchus) blastocysts. *Biology of Reproduction*.

[8] Thomson J. A., Itskovitz-Eldor J., Shapiro S. S., Waknitz M. A., Swiergiel J. J., Marshall V. S., Jones J. M. (1998). Embryonic stem cell lines derived from human blastocysts. *Science*.

[9] Shamblott M. J., Axelman J., Wang S., Bugg E. M., Littlefield J. W., Donovan P. J., Blumenthal P. D., Huggins G. R., Gearhart J. D. (1998). Derivation of pluripotent stem cells from cultured human primordial germ cells. *Proceedings of the National Acadamy of Sciences of the United States of America*.

[10] Andrews PW. (2002). From teratocarcinomas to embryonic stem cells. *Philos Trans R Soc Lond B Biol Sci*.

[11] Klimanskaya I., Chung Y., Becker S., Lu S.-J., Lanza R. (2006). Human embryonic stem cell lines derived from single blastomeres. *Nature*.

[12] Omidi M., Aflatoonian B., Tahajjodi S. S., Khalili M. A. (2019). Attempts for Generation of Embryonic Stem Cells from Human Embryos Following In Vitro Embryo Twinning. *Stem Cells and Development*.

[13] Aflatoonian B., Ruban L., Shamsuddin S., Baker D., Andrews P., Moore H. (2010). Generation of Sheffield (Shef) human embryonic stem cell lines using a microdrop culture system. *In Vitro Cellular & Developmental Biology - Animal*.

[14] Turetsky T., Aizenman E., Gil Y., Weinberg N., Shufaro Y., Revel A., Laufer N., Simon A., Abeliovich D., Reubinoff B. (2007). Laser-assisted derivation of human embryonic stem cell lines from IVF embryos after preimplantation genetic diagnosis. *Human Reproduction*.

[15] Giritharan G., Ilic D., Gormley M., Krtolica A., Cooney A. J. (2011). Human Embryonic Stem Cells Derived from Embryos at Different Stages of Development Share Similar Transcription Profiles. *PLoS ONE*.

[16] Strom S., Inzunza J., Grinnemo K., Holmberg K., Matilainen E., Stromberg A., Blennow E., Hovatta O. (2007). Mechanical isolation of the inner cell mass is effective in derivation of new human embryonic stem cell lines. *Human Reproduction*.

[17] Ilic D., Genbacev O., Krtolica A. (2007). Derivation of hESC from intact blastocysts.. *Current Protocols in Stem Cell Biology*.

[18] Hovatta O., Mikkola M., Gertow K., Strömberg A.-M., Inzunza J., Hreinsson J., Rozell B., Blennow E., Andäng M., Ährlund-Richter L. (2003). A culture system using human foreskin fibroblasts as feeder cells allows production of human embryonic stem cells. *Human Reproduction*.

[19] Desai N., Ludgin J., Goldberg J., Falcone T. (2013). Development of a xeno-free non-contact co- culture system for derivation and maintenance of embryonic stem cells using a novel human endometrial cell line. *Journal of Assisted Reproduction and Genetics*.

[20] Assou S., Pourret E., Péquignot M., Rigau V., Kalatzis V., Aït-Ahmed O., Hamamah S. (2015). Cultured Cells from the Human Oocyte Cumulus Niche Are Efficient Feeders to Propagate Pluripotent Stem Cells. *Stem Cells and Development*.

[21] Adewumi O., Aflatoonian B., Ahrlund-Richter L. (2007). Characterization of human embryonic stem cell lines by the international stem cell initiative. *Nature Biotechnology*.

[22] Miere C., Wood V., Kadeva N., Cornwell G., Codognotto S., Stephenson E., Ilic D. (2016). Generation of KCL037 clinical grade human embryonic stem cell line. *Stem Cell Research*.

[23] Amps K., Jones M., Baker D., Moore H. (2010). In situ cryopreservation of human embryonic stem cells in gas-permeable membrane culture cassettes for high post-thaw yield and good manufacturing practice. *Cryobiology*.

[24] Aflatoonian A., Karimzadeh Maybodi M. A., Aflatoonian N., Tabibnejad N., Amir-Arjmand M. H., Soleimani M., Aflatoonian B., Aflatoonian A. (2016). Perinatal outcome in fresh versus frozen embryo transfer in ART cycles. *International Journal of Reproductive BioMedicine*.

[25] Draper J. S., Pigott C., Thomson J. A., Andrews P. W. (2002). Surface antigens of human embryonic stem cells: changes upon differentiation in culture*. *Journal of Anatomy*.

[26] Sadeghian-Nodoushan F., Aflatoonian R., Borzouie Z., Akyash F., Fesahat F., Soleimani M., Aghajanpour S., Moore H. D., Aflatoonian B. (2016). Pluripotency and differentiation of cells from human testicular sperm extraction: An investigation of cell stemness. *Molecular Reproduction and Development*.

[27] Aflatoonian B. (2016). Human Embryonic Stem Cells Derived Mesenchymal Stem/Stromal Cells and their Use in Regenerative Medicine. *Journal of Stem Cell Research & Therapeutics*.

[28] Aflatoonian B., Moore H.

[29] Laowtammathron C., Chingsuwanrote P., Choavaratana R., Phornwilardsiri S., Sitthirit K., Kaewjunun C., Makemaharn O., Terbto P., Waeteekul S., Lorthongpanich C., U-pratya Y., Srisook P., Kheolamai P., Issaragrisil S. (2018). High-efficiency derivation of human embryonic stem cell lines using a culture system with minimized trophoblast cell proliferation. *Stem Cell Research & Therapy*.

[30] First N., Sims M., Park S., Kent-First M. (1994). Systems for production of calves from cultured bovine embryonic cells. *Reproduction, Fertility and Development*.

[31] Sherbahn R., Frasor J., Radwanska E., Binor Z., Wood-Molo M., Hibner M., Mack S., Rawlins R. G. (1996). Comparison of mouse embryo development in open and microdrop co-culture systems. *Human Reproduction*.

[32] Wakayama S., Hikichi T., Suetsugu R., Sakaide Y., Bui H., Mizutani E., Wakayama T. (2007). Efficient Establishment of Mouse Embryonic Stem Cell Lines from Single Blastomeres and Polar Bodies. *Stem Cells*.

[33] Obruca A., Strohmer H., Sakkas D., Menezo Y., Kogosowski A., Barak Y., Feichtinger W. (1994). Fertilization and early embryology: Use of lasers in assisted fertilization and hatching. *Human Reproduction*.

[34] Veiga A., Sandalinas M., Benkhalifa M., Boada M., Carrera M., Santaló J., Barri P., Ménézo Y. (1997). Laser blastocyst biopsy for preimplantation diagnosis in the human. *Zygote*.

[35] Cortés J., Cobo F., Catalina P., Nieto A., Cabrera C., Montes R., Concha Á., Menendez P. Evaluation of a laser technique to isolate the inner cell mass of murine blastocysts. *Biotechnology and Applied Biochemistry*.

[36] Cortes J. L., Sánchez L., Catalina P., Cobo F., Bueno C., Martínez-Ramirez A., Barroso A., Cabrera C., Ligero G., Montes R., Rubio R., Nieto A., Menendez P. (2008). Whole-blastocyst culture followed by laser drilling technology enhances the efficiency of inner cell mass isolation and embryonic stem cell derivation from good- and poor-quality mouse embryos: New insights for derivation of human embryonic stem cell lines. *Stem Cells and Development*.

[37] Tanaka N., Takeuchi T., Neri Q. V., Sills E. S., Palermo G. D. (2006). Laser-assisted blastocyst dissection and subsequent cultivation of embryonic stem cells in a serum/cell free culture system: Applications and preliminary results in a murine model. *Journal of Translational Medicine*.

[38] Ilic D., Stephenson E., Wood V., Jacquet L., Stevenson D., Petrova A., Kadeva N., Codognotto S., Patel H., Semple M., Cornwell G., Ogilvie C., Braude P. (2012). Derivation and feeder-free propagation of human embryonic stem cells under xeno-free conditions. *Cytotherapy*.

[39] Stephenson E., Jacquet L., Miere C., Wood V., Kadeva N., Cornwell G., Codognotto S., Dajani Y., Braude P., Ilic D. (2012). Derivation and propagation of human embryonic stem cell lines from frozen embryos in an animal product-free environment. *Nature Protocols*.

[40] Strelchenko N., Verlinsky O., Kukharenko V., Verlinsky Y. (2004). Morula-derived human embryonic stem cells. *Reproductive BioMedicine Online*.

[41] Klimanskaya I., Chung Y., Becker S., Lu S., Lanza R. (2007). Derivation of human embryonic stem cells from single blastomeres. *Nature Protocols*.

[42] Chung Y., Klimanskaya I., Becker S., Li T., Maserati M., Lu S., Zdravkovic T., Ilic D., Genbacev O., Fisher S., Krtolica A., Lanza R. (2008). Human Embryonic Stem Cell Lines Generated without Embryo Destruction. *Cell Stem Cell*.

[43] GENBACEV O., KRTOLICA A., ZDRAVKOVIC T., BRUNETTE E., POWELL S., NATH A., CACERES E., MCMASTER M., MCDONAGH S., LI Y. (2005). Serum-free derivation of human embryonic stem cell lines on human placental fibroblast feeders. *Fertility and Sterility*.

[44] Xu C. (2004). Immortalized Fibroblast-Like Cells Derived from Human Embryonic Stem Cells Support Undifferentiated Cell Growth. *Stem Cells*.

[45] Akyash F., Sadeghian-Nodoushan F., Tahajjodi S. S., Nikukar H., Farashahi Yazd E., Azimzadeh M., Moore H. D., Aflatoonian B. (2017). Human embryonic stem cells and good manufacturing practice: Report of a 1- day workshop held at Stem Cell Biology Research Center, Yazd, 27th April 2017. *International Journal of Reproductive BioMedicine*.

